# Outcome of HIV-exposed uninfected children undergoing surgery

**DOI:** 10.1186/1471-2431-11-69

**Published:** 2011-07-29

**Authors:** Jonathan S Karpelowsky, Alastair JW Millar, Nelleke van der Graaf, Guido van Bogerijen, Heather J Zar

**Affiliations:** 1 Department of Pediatric Surgery, Red Cross War Memorial Children's Hospital, School of Child and Adolescent Health, University of Cape Town, South Africa; 2 Department of Pediatric Surgery Erasmus MC-Sophia Children's Hospital, Rotterdam, The Netherlands; 3 Department of Pediatric Medicine, Red Cross War Memorial Children's Hospital, School of Child and Adolescent Health, University of Cape Town, South Africa

## Abstract

**Background:**

HIV-exposed uninfected (HIVe) children are a rapidly growing population that may be at an increased risk of illness compared to HIV-unexposed children (HIVn). The aim of this study was to investigate the morbidity and mortality of HIVe compared to both HIVn and HIV-infected (HIVi) children after a general surgical procedure.

**Methods:**

A prospective study of children less than 60 months of age undergoing general surgery at a paediatric referral hospital from July 2004 to July 2008 inclusive. Children underwent age-definitive HIV testing and were followed up post operatively for the development of complications, length of stay and mortality.

**Results:**

Three hundred and eighty children were enrolled; 4 died and 11 were lost to follow up prior to HIV testing, thus 365 children were included. Of these, 38(10.4%) were HIVe, 245(67.1%) were HIVn and 82(22.5%) were HIVi children.

The overall mortality was low, with 2(5.2%) deaths in the HIVe group, 0 in the HIVn group and 6(7.3%) in the HIVi group (p = 0.0003). HIVe had a longer stay than HIVn children (3 (2-7) vs. 2 (1-4) days p = 0.02). There was no significant difference in length of stay between the HIVe and HIVi groups. HIVe children had a higher rate of complications compared to HIVn children, (9 (23.7%) vs. 14(5.7%) (RR 3.8(2.1-7) p < 0.0001) but a similar rate of complications compared to HIVi children 34 (41.5%) (RR = 0.6 (0.3-1.1) p = 0.06).

**Conclusion:**

HIVe children have a higher risk of developing complications and mortality after surgery compared to HIVn children. However, the risk of complications is lower than that of HIVi children.

## Background

HIV-exposed uninfected (HIVe) children are a rapidly growing population. Programs for the prevention of mother to child transmission (PMTCT) have reduced the transmission rate of perinatal HIV infection to approximately 2% to 5% [1-3]. Such programs have therefore effectively reduced the number of HIV infected (HIVi)children but identified an increasing population of HIVe children [4].

HIVe children have been overlooked as a group of children who may be at an increased risk of illness compared to HIV-unexposed (HIVn) children. Recently, increased morbidity and mortality in HIVe children compared to HIVn children has been reported [4-10]. Many factors may account for this including innate deficiencies in immunity [11-13], feeding practices [14], poor protection from maternal antibodies or environmental exposures [6].

As PMTCT programs expand, an increasing number of HIVe children can be expected to require a routine or emergency surgical procedure [15,16]. Currently no data exist on the risk of morbidity and mortality post-surgery in such children. The aim of this study was to investigate the mortality and post-operative complications in HIVe children compared to both HIVn and HIVi children after a general surgical procedure.

## Methods

A prospective cohort study was performed, from July 2004 to July 2008 at a single tertiary general paediatric surgical centre in Cape Town, Western Cape, South Africa. The general paediatric surgical service acts as a regional and national referral centre with approximately 2400 operations performed annually. The study was approved by the ethics committee of the Faculty of Health sciences, University of Cape Town.

The study site is a high HIV prevalence area, with an estimated HIV prevalence amongst pregnant women of approximately 16% [17]. There is a well-developed PMTCT program with HIV transmission rates of approximately 2-4%. The Western Cape has approximately 70000 births per annum [1], of which approximately 11000 babies per year are expected to be HIVe.

Inclusion criteria were children less than 60 months of age, undergoing a general surgical procedure. The recruitment was conducted in two phases. 1) Phase 1(pilot data), enrolled only HIV-exposed children, from July 2004 to December 2006. This was done to gather pilot data, to inform the larger phase 2 study. 2) Phase 2 enrolled all children irrespective of HIV exposure from January 2007 until July 2008 inclusive. Analysis was conducted on both phase 1 and phase 2 data.

Age definitive HIV testing with pre and post-test counselling was done in children whose HIV status was unknown. HIV infection was defined as 2 positive HIV ELISA (Determine^®^Abbott, Abbott Park, Ill. USA) tests in children > 18 months; a positive ELISA test, and if positive a confirmatory PCR test in children less than 18 months. HIV exposure was defined as a positive ELISA but negative PCR in children < 18 months or in children > 18 months knowledge of an infected mother but a negative ELISA in the child.

Informed consent from a parent or legal guardian was obtained. Details of the child's demographics, clinical examination, medication, haematological tests, HIV status and treatment, surgery and complications were recorded until discharge, death or in the case of prolonged admissions 60 days following their last surgical procedure or surgical complication.

Children formed 3 groups- 1. HIV-exposed (HIVe) but uninfected 2.HIV unexposed (HIVn), 3. HIV infected (HIVi). HIVe children were compared to the HIVn and the HIVi groups

A procedure entailed a general surgical operation under general anaesthetic. Surgical procedures were defined as emergency (which in the opinion of the treating clinician unless performed in 24 hours of hours of admission would result in either morbidity or mortality of the child) or elective. Procedures were also defined as major (which entailed entry into a body cavity (abdomen or thorax), oncological resection, and a prolonged procedure longer than 90 min or vascular reconstruction) or minor. A contaminated case was defined as pus, infection, or gross contamination present at the site of surgery when performing the procedure. Prophylactic antibiotics were used in major cases or contaminated cases according to local microbiological guidelines. This included a cephalosporin and metronidazole or alternatively, penicillin an aminoglycoside and metronidazole, depending on the procedure.

The primary outcome was the occurrence of a complication, defined as an adverse event occurring in the intra, or post-operative period which is not expected to occur either during or following the normal course of the procedure. Secondary outcomes were length of stay (calculated in days from the day of admission until the day of discharge or death) and mortality.

### Statistical analysis

Descriptive data is presented as means +/- standard deviation, medians with an interquartile range (IQR) or proportions with a 95% confidence interval. Normality of data was tested using the Shapiro-Wilks test. Hypothesis testing, using either the chi squared or fisher exact test for dichotomous or ordinal variables was done. The student t-test was used for the comparison of means and the Wilcoxon rank-sum test for comparing medians of continuous variables. Nutrition assessed using weight for height Z scores as a continuous variable and a dichotomous variable with a weight for age Z-Score of less than -2 being malnourished. Relative risks were calculated for complications or mortality. Following the initial comparisons, age adjusted control groups were formed in the HIVn and HIVi groups due to the significantly younger age of the HIVe children. This was done by selecting appropriate matched controls within one month of age for each HIVe case. Due to the sample size, 3 age matched controls could be selected from the HIVn group, and a single age matched control from the HIVi group. Furthermore age as a confounding variable was tested on multivariate analysis in three different models, the entire cohort, HIVe and HIVn children and HIVe and HIVi children. The regression models were constructed for complications. This was done by including factors significant on univariate analysis or alternatively of strong clinical importance as a backward step regression until the best fit was obtained. Data was analysed using Stata (Version10.0, College Station, Tx USA). Significance was set at p < 0.05 and results were expressed as proportions and relative risk with 95% confidence intervals.

## Results

Three hundred and eighty children were enrolled (94 during, phase 1); 4 died and 11 were lost to follow up prior to PCR testing, thus 365 children were included. Of these, 38(10.4%) were HIVe, 245(67.1%) were HIVn and 82(22.5%) were HIVi children.

The median age of children was 11 (IQR 4.4-26.6) months. HIVe children were younger, 4.5 (IQR 0.8-11.2) months than the HIVi (11.5 months (IQR 6-24) p < 0.0001) or HIVn (12.7 months (IQR 4.4-29.4) p = < 0.0001) children. HIVe children had a higher number of major procedures, contamination at the surgical site and worse nutrition compared to HIVn children (table 1). Following age matching of HIVe and HIVn children, an increased surgical site contamination and worse nutrition persisted in HIVe children (table 2). HIVe and HIVi children were well matched for the level of procedure, surgical site contamination, urgency, nutrition and number of urgent procedures in both the age matched and unmatched groups (table 3, 4)

**Table 1:** Comparison of HIV exposed uninfected and HIV unexposed children

	HIVe(n = 38)	HIVn(n = 245)	Relative Risk	Significance
**Age(months)**	4.5 (0.8-11.2)	12.7(4.4-29.4)		p < 0.00001*
**Nutrition**				
• Z-score weight for age	-1.74 (-3.4- -0.5)	-0.63(-1.7-0.18)		P = 0.0002*
• Malnutrition (Z-score<-2)	18 (47.4%)	43 (17.5%)	RR = 2.7(1.8-4.2)	p < 0.00001*
**Major cases**	17 (44.7%)	64 (26.1%)	RR = 1.7(1.2-2.6)	P = 0.01*
**Contaminated cases**	15 (39.4%)	49 (20%)	RR = 2 (1.3-3.1)	P = 0.008*
**Emergency cases**	12 (31.5%)	95 (38.8%)		P = 0.8
**No. of patients with a Complication**	9 (23.7%)	14 (5.7%)	RR = 4.2 (1.9-8.9)	p < 0-.0001*
**Mortality**	2 (5.2%)	0(0%)		P = 0.0003*
**Length of stay (days)**	3-(2-7)	2(1-4)		P = 0.02*

Values expressed as medians (interquartile range) for continuous and number (percentage) for dichotomous variables. Relative risk is expressed as the RR (95%confidence interval) * are significant values.

**Table 2:** Comparison HIV exposed uninfected children and age matched HIV unexposed children

	HIVe(n = 38)	Age matched HIVn(n = 114)	Relative Risk	Significance
**Age(months)**	4.5 (0.8-11.2)	4.4(1.8-11)		p = 0.6
**Nutrition**				
• Z-score weight for age	-1.74 (-3.4- -0.5)	-0.86(-1.8- 0)		P = 0.01*
• Malnutrition (Z-score<-2)	18 (47.4%)	26 (22.8%)	RR = 2 (1.3-3.3)	p = 0.004*
**Major cases**	17 (44.7%)	34(29.8%)		p = 0.09
**Contaminated cases**	15 (39.4%)	20(17.5%)	RR = 2.1 (1.3-3.7)	p = 0.01*
**Emergency cases**	12 (31.5%)	51(44.7%)		p = 0.2
**No. of patients with a Complication**	9 (23.7%)	10(8.7%)	RR = 2.3 (1.3-4.1)	p = 0.008*
**Mortality**	2 (5.2%)	0(0)		p = 0.01*
**Length of stay (days)**	3-(2-7)	2(1-5)		P = 0.2

Values expressed as medians (interquartile range) for continuous and number (percentage) for dichotomous variables. Relative risk is expressed as the RR (95%confidence interval) * are significant values.

**Table 3:** Comparison of HIV exposed uninfected and HIV infected children

	HIVe(n = 38)	HIVi(n = 82)	Significance
**Age(months)**	4.5 (0.8-11.2)	11.5(6-24)	p < 0.0001*
**Nutrition**			
• Z-score weight for age	-1.74 (-3.4- -0.5)	-2.0(-3.4- -0.97)	P = 0.7
• Malnutrition (Z-score<-2)	18 (47.4%)	41 (50.0%)	P = 0.8
**Major cases**	17 (44.7%)	28 (34.2%)	P = 0.1
**Contaminated cases**	15 (39.4%)	40 (48.7%)	P = 0.2
**Emergency cases**	12 (31.5%)	37 (45.1%)	P = 0.8
**No. of patients with a Complication**	9 (23.7%)	34 (41.5%)	P = 0.06
**Mortality**	2 (5.2%)	6 (7.3%)	P = 0.8
**Length of stay (days)**	3-(2-7)	4(2-14)	P = 0.5

Values expressed as medians (interquartile range) for continuous and number (percentage) for dichotomous variables. Relative risk is expressed as the RR (95%confidence interval) * are significant values.

**Table 4:** Comparison HIV exposed uninfected and age matched HIV infected children

	HIVe(n = 38)	Age matched HIVi(n = 38)	Relative Risk	Significance
**Age(months)**	4.5 (0.8-11.2)	6(4.4-10)		p = 0.4
**Nutrition**				
• Z-score weight for age	-1.74 (-3.4- -0.5)	-1.9(+/-1.7)		P = 0.9
• Malnutrition (Z-score<-2)	18 (47.4%)	19 (50%)		P = 0.8
**Major cases**	17 (44.7%)	16 (42.1%)		P = 0.8
**Contaminated cases**	15 (39.4%)	20(52.6%)		P = 0.2
**Emergency cases**	12 (31.5%)	14 (36.8%)		P = 0.6
**No. of patients with a Complication**	9 (23.7%)	24 (63.2%)	RR = 0.40 (0.22-0.73)	P = 0.0005*
**Mortality**	2 (5.2%)	5 (13.1%)		P = 0.4
**Length of stay (days)**	3-(2-7)	4(2-22)		P = 0.2

Values expressed as medians (interquartile range) for continuous and number (percentage) for dichotomous variables. Relative risk is expressed as the RR (95%confidence interval) * are significant values.

The overall mortality (8 deaths, 2.2%) was low, with 2(5.2%) deaths in the HIVe group, none in the HIVn group and 6(7.3%) in the HIVi group. The mortality rate was significantly higher in HIVe compared to HIVn children (p = 0.0003). This difference persisted when matching for age p = 0.01 (table 2). No significant difference in mortality was demonstrated between HIVe and HIVi children (table 3, 4).

HIVe had a longer duration of hospitalisation than HIVn children (3 (2-7) vs. 2 (1-4) days p = 0.02), but this difference did not persist with age matching of the groups ((3 (2-7) vs. 2 (1-5) days p = 0.2). There was no significant difference in length of stay between the HIVe and HIVi groups.

Overall 57 (15.6%) children developed a complication. The HIVe children had a higher rate of complications, compared to HIVn children [9 (23.7%) vs. 14(5.7%) (RR 4.18(1.9-8.9) p < 0.0001)] (table 1), but a similar rate compared to HIVi children [9(23.7%) vs. 34 (41.5%) (RR = 0.6 (0.3-1.1) p = 0.06)] (table 3). Using aged matched controls there was still a significant difference in the rate of complications between HIVe and HIVn children [9 (23.7%) vs. 10 (8.7%) (RR 2.3 (1.3-4.1) p = 0.008)] (table 2). After age matching, HIVe children had a lower risk of complications than HIVi children [(9(23.7%) vs. 24 (63.2%)(RR 0.4 (0.2-0.7) p = 0.0005)] (table 4)

A total of 71 complications occurred in 57 children (table 5). The commonest complications were surgical site complications in 34/71 (48%). Of these 13/34(38%) were wound infection, 7/34(20.5%) were wound breakdown (without clinical or microbiological infection), 7/34(20.5%) involved breakdown of an enteric stoma, 4/34(11%) were early recurrence of the surgically treated condition and 3/34(9%) were anastomotic obstruction or stenosis. The second commonest complication was post-operative systemic infection, comprising pneumonia in 10/31(32%), sepsis with bacteraemia in 13/31(42%), central line sepsis 5/31(16%) and one case each of cholangitis, urinary infection and intra-abdominal sepsis 3/31(10%). Other complications occurring in 6/71(8%) included postoperative airway obstruction due to vocal cord palsy, dehydration and hypoglycaemia. Of the complications recorded, HIVe children had a higher proportion of systemic post-operative infections than both HIVn children [12/16(75%) vs. 8/18(44%) p = 0.05 RR 1.7 (1.1-3)] and HIVi children [12/16(75%) vs. 11/37(30%) p = 0.002 RR = 2.5(1.4-4.5)]

**Table 5:** Comparison of post-operative complications in HIV infected, HIV unexposed and HIV exposed uninfected children

	HIV Status		
**Total Complications **(71)	**HIVi **(37)	**HIVn **(18)	**HIVe **(16)

Systemic Post-operative infection 31/71(44%)	11/37 (30%)	8/18 (44%)	12/16(75%)
Pneumonia	5/11(45.5%)	2/8(25%)	3/12(25%)
Bacteraemia	5/11(45.5%)	2/8(25%)	6/12(50%)
Central Line sepsis	1/11(9%)	2/8(25%)	2/12(16.7%)
Urinary tract	0	1/8 (12.5%)	0
Cholangitis	0	1/8 (12.5%)	0
Intra-abdominal	0	0	1/12 (8.3%)
Surgical site complication 34/71(48%)	24/37(65%)	6/18(33%)	4/16 (25%)
Wound Infection	8/24(33%)	3/6(50%)	2/4(50%)
Wound break down	7/24(29%)	0	0
Stoma complication	5/24(21%)	1/6(17%)	1/4(25%)
Recurrence	4/24(17%)	0	0
Anastomotic obstruction/stenosis	0	2/6(33%)	1/4(25%)
			
Miscellaneous 6/71 (8%)	2/37(5%)	4/18(22%)	0

Logistic regression comparing HIVe and HIVn children and HIVe and HIVi children included significant variables on univariate analysis, and other clinically important variables such as nutrition, urgency of surgery and surgical site contamination. Following backward step regression for HIVe and HIVn children it was demonstrated that HIV exposure and undergoing a major procedure were the most predictive of developing a complication (table 6). Models for HIVe and HIVi children demonstrated that HIV status, age under 12 months and undergoing a major procedure were the most predictive of developing a complication (table 7).

**Table 6:** Logistic regression for complications with HIV exposed uninfected children and HIV unexposed children

Variable	Odds Ratio	Significance	95% confidence interval
HIV exposure	4.9	P = 0.006	1.6-15.4
Age	0.9	P = 0.09	0.9-1.2
Major procedure	9.2	p < 0.0001	3.2-25.0

**Table 7:** Logistic regression for complications with HIV exposed uninfected children and HIV infected children

Variable	Odds Ratio	Significance	95% confidence interval
HIV exposure	0.5	p = 0.01	0.3-0.8
Age under 12 months	5.2	p = 0.001	1.9-14.4
Major procedure	5.5	p < 0.0001	2.1-14.0

## Discussion

This study has shown that HIVe children undergoing surgery have an intermediate risk of complications post-surgery; higher than that of HIVn children, but lower than HIV infected HIVi children. In addition, in hospital mortality post-surgery was higher in HIVe children compared to that in HIVn children.

The increased morbidity and mortality related to HIV exposure may be due to many factors. HIVe children may have poorer growth and nutrition, increased risk of infection, impaired passive immunity from maternal antibodies, or greater exposure to potential pathogens in a household with a HIV infected adult.

HIVe children had worse nutrition compared to those who were HIVn. Reasons for poorer nutrition may include parental illness and poverty [18,19], infant feeding practices [14,18], lack of breast feeding, and an increase disease burden in exposed children [6,13,14,19]. Although breast feeding rates were not recorded, it is likely that there would have been a low breast feeding rates in keeping with the PMTCT policy, which was formula feeding of children born to HIV-infected mothers in the Western Cape Province at the time of the study. Several cohort studies have reported HIVe children to have worse nutrition compared to HIVn children [19].

HIVe children have been reported to have an increased risk of infection compared to HIVn children [4,6,10,19-23]. Infections in HIVe also tend to be more severe than in HIVn children [6,10,19,24,25]. Many of these infections are opportunistic infections occurring in an immune-compromised host [4,10,19,24]. There appears that in addition to environmental and feeding practices HIVe children have an impaired innate immunity [5,19]. This may increase susceptibility to infection and increase the risk for developing post-operative complications. This was consistent with our findings of a high rate of infections in HIVe children post operatively.

Major procedures are a clearly identified risk factor associated with an increased risk for the development of complications [26] and risk stratification to account for complications is well described [27,28]. Increased complexity of the procedure (thus increasing the risk of technical problems), immune dysregulation related to the stress response to surgery or impaired respiratory function in the post-operative period may all contribute to this [29].

There are several limitations to this study. The relatively lower age of the HIVe compared with HIVi and HIVn patients may have been a confounding variable as the HIVe children may not have had a fully developed immune system. We have attempted to account for this in multivariate analysis and secondly by age matching to HIVi and HIVn children. In both instances when age was accounted for, HIV status had an important effect on post-operative outcomes with HIVe children having a risk in between that of HIVn and HIVi children. This study has a small number of HIVe children which may have influenced the results and larger studies are needed with stratification of the HIVe group based on age of the child, feeding practice and the stage of maternal HIV disease. Lastly as this was an observational cohort study HIV results were not blinded to either treating physicians or observers and this may have resulted in some reporting bias.

## Conclusion

This is the first study to report that HIVe children undergoing surgery have a higher risk of developing complications and mortality compared to HIVn children, but a lower risk than that of HIVi children. This should be considered when assessing the risk benefit ratio and age of any surgical procedure.

## Competing interests

The authors declare that they have no competing interests.

## Authors' contributions

JSK - designed the study, protocol, and case record folders (CRF) as well as performed the analysis. He wrote the draft manuscript and incorporated comments from co-authors. The ethics application and funding applications were submitted by JK as the principal investigator. NvG; GvB- Collected data and data entry as well as follow up of patient data AJWM; HJZ- contributed to the study design, analysis and writing of the manuscript. All authors have read and approved the manuscript.

## Pre-publication history

The pre-publication history for this paper can be accessed here:

http://www.biomedcentral.com/1471-2431/11/69/prepub
